# Uncertainty in Population Growth Rates: Determining Confidence Intervals from Point Estimates of Parameters

**DOI:** 10.1371/journal.pone.0013628

**Published:** 2010-10-25

**Authors:** Eleanor S. Devenish Nelson, Stephen Harris, Carl D. Soulsbury, Shane A. Richards, Philip A. Stephens

**Affiliations:** 1 School of Biological and Biomedical Sciences, Durham University, Durham, United Kingdom; 2 School of Biological Sciences, University of Bristol, Bristol, United Kingdom; University of Fribourg, Switzerland

## Abstract

**Background:**

Demographic models are widely used in conservation and management, and their parameterisation often relies on data collected for other purposes. When underlying data lack clear indications of associated uncertainty, modellers often fail to account for that uncertainty in model outputs, such as estimates of population growth.

**Methodology/Principal Findings:**

We applied a likelihood approach to infer uncertainty retrospectively from point estimates of vital rates. Combining this with resampling techniques and projection modelling, we show that confidence intervals for population growth estimates are easy to derive. We used similar techniques to examine the effects of sample size on uncertainty. Our approach is illustrated using data on the red fox, *Vulpes vulpes*, a predator of ecological and cultural importance, and the most widespread extant terrestrial mammal. We show that uncertainty surrounding estimated population growth rates can be high, even for relatively well-studied populations. Halving that uncertainty typically requires a quadrupling of sampling effort.

**Conclusions/Significance:**

Our results compel caution when comparing demographic trends between populations without accounting for uncertainty. Our methods will be widely applicable to demographic studies of many species.

## Introduction

Demographic modelling is widely used in conservation and management [1], [2]. As modelling techniques have become increasingly sophisticated, a growing literature has dealt with the importance of acknowledging process error (or environmentally-driven variation in demographic parameters) in model analyses [3], [4], [5]. By contrast, assessments of the implications of observation error (arising from sampling limitations) for model precision are often lacking, but see [6], [7], perhaps due to a widespread acknowledgement of the ubiquity of sampling constraints [8]. Here, we discuss methods to infer accuracy of vital rate estimates, even where parameter uncertainty has not been reported explicitly. We show that acknowledging limits to precision can be an important element of demographic inference, with implications for data collection protocols.

Age- or stage-structured (Leslie or Lefkovitch) matrix population models are conceptually clear and relatively easily parameterised, with well-characterised properties; as such, the use of matrix models is particularly widespread in ecology [4], [9]. Studies utilising matrix population modelling rely on data from a variety of sources. Frequently, the studies' authors have also collected the demographic data used to parameterise the transition matrix. In these cases, sample variance is used to establish vital rate distributions and resampling techniques are available to determine the consequences of that uncertainty for estimates of population growth e.g., [10], [11]. In spite of this, many authors routinely publish point estimates of asymptotic population growth (λ), without accompanying metrics of precision such as standard errors or confidence intervals. Furthermore, this practice is not limited to relatively low-ranking journals; see supplementary material, Table S1.

When modellers use data that were not collected specifically for the purposes of demographic insight, further problems arise. Hunting records are a common source of such data, even though they are associated with a number of important assumptions that limit their use and compel caution in their interpretation [12]. Even accepting these limitations, hunting data are often reported inconsistently and, in particular, are frequently presented without estimates of accompanying uncertainty. In these situations, likelihood approaches provide a convenient method to infer the distribution and extent of uncertainty around the best estimate for the parameter of interest. Hitherto, likelihood methods have largely been neglected for exposing the uncertainty associated with the output of projection matrices.

In this paper, we present techniques for inferring, retrospectively, the uncertainty of demographic parameters due to observation error in demographic data. Following others e.g., [13] we distinguish between Bernoulli processes, such as survival or probability of breeding, and Poisson processes, such as litter size. We illustrate this approach with reference to the red fox (*Vulpes vulpes*), the most widely distributed extant wild terrestrial mammalian species [14], extensively studied throughout its geographic range due to its ecological, economic, and cultural importance e.g., [15], [16]. The red fox is widely hunted, making the species a rich source of demographic data. Comparisons of red fox population growth rates in different parts of the world have been used to classify the species along the “fast-slow” life history continuum [17], and have also been used to make inferences about the species' response to different environmental and management pressures [18]. Determining the confidence that we can place in these assessments is, therefore, crucial for a number of applications.

Here, we begin by illustrating how likelihood profiles can be determined for red fox demographic parameters and use resampling techniques to assess confidence in resultant estimates of population growth. Our results highlight the need for caution in generalising about differences in the dynamics of populations. We illustrate the utility of this resampling approach to provide information about required sampling effort.

## Methods

### Likelihood profiles for demographic parameters

Age-specific survival and proportion of breeding females are Bernoulli processes, in the sense that each female can be considered a ‘trial’ with a binomial outcome (live or die, breed or fail to breed). Taking the example of survival, hunting data often yield numbers of individuals in different age classes. If the data are assumed to have been collected at a time when the population approximated its stable age distribution, survival of individuals of age *x* can be inferred from the relative number of individuals in age classes *x* and *x*+1 (*f_x_* and *f_x_*_+1_, respectively). The point estimate of survival, *P_x_*, is given by *P_x_* = *f_x_*_+1_/*f_x_*. Occasionally, *f_x_*_+1_>*f_x_*, or the population is known to have been growing at some rate (*r*) during the period of data collection; Caughley [12] presents methods to deal with both of these situations. Very often, sample sizes for older age classes are sufficiently restricted that it is useful to truncate the age distribution and create composite classes for all age classes beyond a given age. In these cases, the point estimate of survival is given by P_x__*_ = *f_x_*_>x*_/ (*f_x_*+*f_x_*_>x*_), where x* is the final age class.

In the previous formulae, the number of trials is represented by the denominator of the point estimate equation, whilst the number of events (or successes) is given by the numerator. However, the point estimate for survival is only an estimate. It is often more interesting to consider the relative probability with which any other true parameter value could have yielded the same outcome, i.e. the same number of events from the same number of trials. Assuming a uniform prior probability for any putative survival rate, the likelihood of any given survival rate, *P_x_*, is given by: 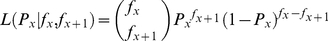
(1)


This likelihood distribution is easily evaluated using the “dbinom(events,trials,*P_x_*)” function in R. Given data, for example, on the proportion of shot females that show signs of breeding, the same approach can be used to determine the likelihood profile for the probability of breeding, *B_x_*. If we have prior information about the focal parameter, then it can easily be incorporated using a Bayesian approach [13].

When estimating age-dependent, per capita, fecundity rates we assume that we only have information on the number of females of age *x* that bred, denoted *N_x_*, and the total number of offspring that they produced, denoted *Y_x_*. Here, we assume that the number of offspring a female produces, given that she has produced at least one offspring, is distributed according to a shifted Poisson distribution. The point estimate for average litter size for breeding females in age class *x* is simply *m_x_* = *Y_x_*/*N_x_*. The likelihood that the true mean litter size is *m_x_*, is:
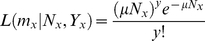
(2)where *µ* = *m_x_*−1 and *y* = *Y_x_*−*N_x_*. These adjustments are necessary to shift the Poisson distribution of litter sizes one interval to the right [19], removing the possibility of zero litter sizes for females that breed. This likelihood distribution is also easily determined in R using the “dpois(*y*, *µN_x_*)” function.

#### Confidence intervals for population growth estimates: the red fox as an example

We extracted published demographic data for three red fox populations of management interest: a culled Australian population [20], [21], [22], a non-culled Australian population [23], and a culled USA population [24], [25], [26], [27], [28]. We constructed female-only, post-breeding ‘birth-pulse’ models of the form **N** *_t_*_+1_ = **A**_._ **N** *_t_*, where **N** *_t_* is a vector of numbers of females in each age class at time *t* and **A** is the transition matrix. The transition matrix was based on four age classes (juveniles, <1 year; yearlings, 1–2 years; young adults, 2–3 years; and older adults, ≥3 years) and took the form:
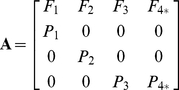
(3)


To avoid small sample size issues among older age classes, only four age classes were used; it is unusual for individuals to survive past 4 years [25], [29].

Deterministic growth, λ*_i_*, of population *i*, was determined from the dominant eigenvalue of **A** *_i_* using point estimates of each matrix element for survival, calculated as detailed above. Fecundity matrix elements (*F_x_*) were determined from the proportion of breeding females (*B_x_*), the average age-specific litter size (*m_x_*) and a generalised birth sex ratio of 1∶1 [30], so that F_x_ = 0.5*P_x_B_x_m_x_*.

Confidence intervals were determined using a resampling (or parametric bootstrap) approach [10]. Specifically, we determined λ*_i_* from 10,000 replicate projection matrices, with each element drawn from its corresponding likelihood distribution; confidence intervals for λ*_i_* were taken as the range encompassing the central 95% of λ*_i_* estimates.

### Implications for sample size

To illustrate an additional benefit of the resampling approach for quantifying uncertainty, we created a ‘generic’ red fox population, sensu [31], from the focal studies. Generic demographic parameters were calculated by summing ‘events’ and ‘trials’ across the three studies; thus, parameters were weighted by the size of studies. The stable stage distribution (SSD) was calculated from the right eigenvector of the generic projection matrix, **A** *_g_*. We investigated the effect of different sample sizes on the level of confidence that could be placed in estimates of population growth, λ*_g_*. Specifically, for a given sample size, *S*, we assumed that the number of females available for demographic analysis was proportioned among age classes according to the SSD. We selected those *S* individuals randomly, resampling with replacement, and calculated all matrix elements according to the fates of the selected individuals (whether they lived or died, bred or failed to breed and, if they bred, the number of offspring they produced, drawn from the relevant likelihood distribution). From this resampled matrix, we determined λ*_g,S,j_*, where *S* was the sample size and *j* = 1, 2 … 10^4^ resampled matrices. We repeated the process for a range of sample sizes from 50 to 2,000 females, reflecting the range of sample sizes available for published studies of red foxes (minimum 42 [28], maximum 1701 [32]). Resultant 95% confidence intervals for estimates of λ*_g,S_* were plotted against sample size.

## Results

### Red fox demographic parameters

Demographic parameters for the three focal populations are summarised in Table 1. Also shown are the parameters for the generic population, derived by combining data from the three studies.

**Table 1:** Demographic data used to define projection matrices for three independent red fox populations and a ‘generic’ population based on data from the three other populations.

Parameter	Notation	Australia*	Australia† (non hunted)	USA‡	Generic * † ‡
Age distribution	*f_0_*	518	51	1992	2561
	*f_1_*	143	20	817	980
	*f_2_*	88	13	216	317
	*f_3_*	67	14	168	249
	*f_4_* *	32	3	62	97
Survival *f_x+1_/f_x_*	*P_1_*	0.28	0.39	0.41	0.38
	*P_2_*	0.62	0.65	0.26	0.32
	*P_3_*	0.53	0.92	0.60	0.79
	*P_4_*	0.32	0.18	0.27	0.28
Probability of breeding (sample size)	*B_1_*	0.77 (200)	1.00 (19)	0.68 (82)	0.76 (301)
	*B_2_*	0.88 (64)	1.00(13)	0.92 (36)	0.90 (113)
	*B_3_*	0.88 (34)	1.00 (9)	0.91 (22)	0.91 (65)
	*B_4_*	0.94 (54)	1.00 (3)	0.97 (34)	0.96 (91)
Mean litter size (sample size)	*m_1_*	3.22 (154)	3.50 (19)	4.52 (73)	3.75 (246)
	*m_2_*	4.00 (56)	3.91 (13)	5.07 (35)	4.33 (104)
	*m_3_*	4.80 (30)	3.09 (9)	5.83 (21)	4.57 (60)
	*m_4_*	4.80 (51)	3.76 (3)	5.91 (33)	4.82 (87)
Fecundity 0.5*P_x_ B_x_m_x_*	*F_1_*	0.34	0.69	0.63	0.55
	*F_2_*	1.08	1.27	0.61	0.63
	*F_3_*	1.13	1.43	1.59	1.63
	*F_4_*	0.73	0.33	0.77	0.65

*[20], [21], [22];

† [23]:

‡ [24], [25], [26], [27], [28]. Sample sizes in parenthesis.

### Likelihood profiles for demographic parameters

**Figure 1:**
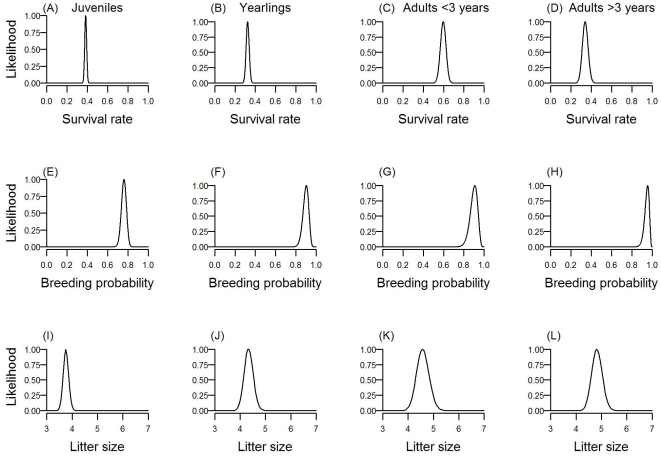
Likelihood distributions for demographic parameters of the hunted Australian population. From left to right for age classes 1 to 4.: (a–d) survival rates (*P_x_*), (e–h) probability of breeding (*B_x_*) and (i–l) litter size (*m_x_*). All likelihoods were rescaled to peak at 1.0.

The width (or, equivalently, uncertainty) of likelihood distributions is clearly influenced by both sample size and mean survival rate. Uncertainty is greatest for intermediate vital rates (e.g. probabilities closer to 0.5 than to either zero or unity) and when sample size is low (see Figure S1 in supporting information). Likelihood profiles were determined for each of the demographic parameters: an example for the Australian population is shown in Figure 1. The SSD for these populations is heavily skewed towards younger age classes and this is reflected in the sample sizes available for each age class (see Table 1); hence, there is a tendency for likelihoods to show wider distributions for all parameters associated with older age classes (Figure 1). The exception to this is the final age class, at which the age distribution is truncated, which has the potential for larger sample sizes than the penultimate age class.

### Confidence intervals for population growth estimates

**Figure 2:**
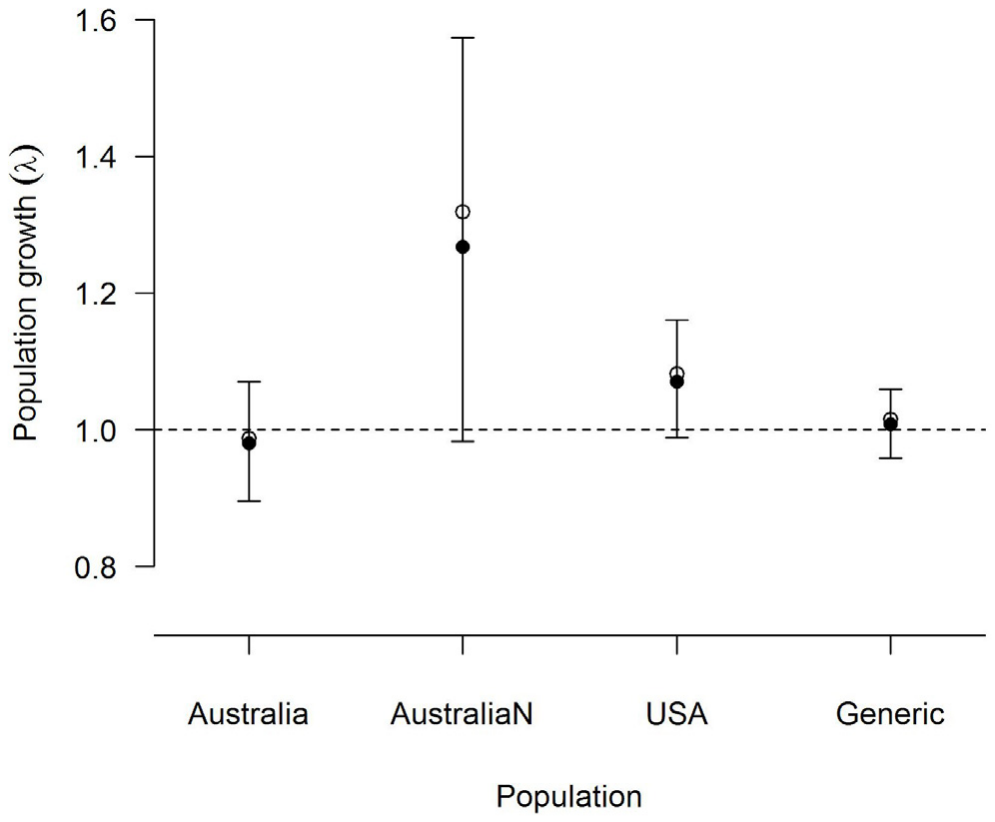
Asymptotic population growth rates (λ) for three red fox populations and a ‘generic’ population. The figure shows point estimates, determined from the dominant eigenvalue of the population's projection matrix (open circles), as well as mean (filled circles) and 95% confidence intervals (error bars) determined from 10^4^ Monte Carlo resamples from the likelihood distributions of all underlying parameters. The line at λ = 1 indicates stability.

Confidence intervals associated with population growth estimates were generally large and all overlapped with λ = 1, denoting a stable population (Figure 2). That all the confidence intervals overlapped with unity does not suggest that these are likely to be stable populations, but it does highlight the uncertainty arising from observation error alone. For example, the point estimate of population growth for the relatively intensively studied USA population (with survival data inferred from over 3,000 culled foxes from the combined studies) suggested an annual increase of approximately 8%. By contrast, 95% confidence intervals for that population varied from suggesting a decline of over 1% per annum, to an annual increase of nearly 16%. Ignoring density dependence, this range of outcomes is equivalent to a population that could decline by 10% over 7 years, to one that could grow by 100% in just 5 years.

In each case, the point estimate of λ was slightly higher than the stochastic mean estimate. This is particularly noticeable for the non-hunted Australian population, which has very small sample sizes. This overestimation can be explained by Jensen's inequality, a mathematical property of non-linear functions. Specifically, the overestimation will occur if lambda is a non-linear decelerating function of a given parameter [7].

### Implications for sampling effort

**Figure 3:**
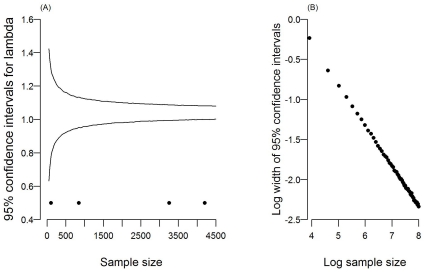
Effect of sample size on uncertainty associated with estimated asymptotic population growth (λ). (a) 95% confidence intervals were calculated by resampling with replacement from the individuals available from the generic population (see text for further details). Letters indicate total sample sizes for the age distributions of the focal populations [A(N), non-hunted Australia = 101; A, Australia = 848; U, USA = 3255; G, Generic = 4204]. (b) Log sample size plotted against log of the width of the 95% confidence intervals (slope = −0.50).

Confidence intervals around estimates of the generic population's asymptotic growth rate were initially broad but reduced in width at a decreasing rate as sample size increased (Figure 3). In fact, in line with probability theory, the width of the 95% confidence intervals declines with sample size to the half power (Figure 3b), indicating that to reduce confidence intervals by half, the sample size needs to be increased fourfold.

## Discussion

Matrix modelling [4], resampling techniques [10] and likelihood approaches [33] are increasingly commonly used in ecology. In spite of this, they do not appear to have been combined to provide insight into the limitations imposed on matrix projections by observation error. Here, we have shown that deriving likelihood distributions from point estimates of demographic parameters is straightforward with freely-available software. Resampling from parameter distributions derived from published studies of red fox demography and focusing on estimates of asymptotic growth rate, we have illustrated that observation error can introduce substantial uncertainty into demographic inference. Our results have implications when applying matrix projection models to real world problems and, more generally, for the interpretation of demographic data.

Resampling from measured distributions has been applied to matrix projection models to determine the impacts of a range of sources of variation on the population dynamics of the focal system e.g., [11], [34]. However, when matrix parameters are derived from data from other sources, accompanying measures of uncertainty are often lacking. This is particularly the case when data are derived from hunting records. Indeed, even in situations in which raw data are available, our review of published studies suggests that generating point estimates of asymptotic growth rate without accompanying estimates of uncertainty is common practice (Table S1 in supporting information). In either case, our example shows that it should be perfectly possible to infer uncertainty in underlying parameters and, using resampling, to assess how that uncertainty propagates through to insights into population dynamics. This is particularly pertinent, given that demographic models are frequently relied upon to make predictions based on these ‘uncertain’ estimates, such as with regard to sustainable harvesting rates [31] and minimum viable population sizes [35].

Recently, concerns have emerged regarding an over-reliance on stable, asymptotic properties of projection matrices. Ezard *et al.* [9] noted that anthropogenic impacts frequently perturb populations away from their expected stable stage distributions (SSD), with the result that transient dynamics following a disturbance can depart significantly from the dynamics associated with asymptotic conditions. The consequence is that longer term trajectories can be quite different from those predicted by standard deterministic projection matrix analyses. Ezard *et al.* [9] recommended a greater focus on transient dynamics and, in particular, a focus on matrix properties decoupled from the assumption of SSDs e.g. by using analyses based on observed stage distributions. We also urge caution in the interpretation of dynamical parameters derived from standard matrix projection analyses. Indeed, our resampling of likelihoods approach could easily be combined with Ezard *et al.* 's [9] focus on observed stage distributions.

Although we present likelihood methods as a useful way to infer uncertainty in point estimates of demographic parameters, we consider this a starting point for more critical analyses of demographic data. For example, McCarthy [13] presents methods for improving the construction of parameter likelihoods through the establishment of informative priors. In addition, although we used a shifted Poisson distribution to describe litter sizes, further analyses are required to identify the most generally applicable distributions. In the specific case of litter size, Morris and Doak [36] have suggested that the stretched beta might be more appropriate. In general, we advocate a greater use of online supplementary materials to provide raw data emerging from studies, in order to aid future analyses of vital rate distributions. In this context it is also important for age distributions to be presented as yearly data (rather than aggregated across years) to improve estimations of vital rate variance, construct periodic models, and incorporate stochasticity.

Red foxes are widespread and often abundant and, as a result, they have been extensively studied in a wide range of locations. In spite of this, it remains the case that most fox demographic data are collected through hunting returns. Utilising these minimal data to their maximum potential is important, not only for foxes, but for other species for which demographic data are collected by similar methods e.g., [37], [38], [39]. Increasing our understanding of fox population dynamics is important for designing more efficient management strategies, predicting effects of environmental changes, and understanding evolutionary processes. Although several studies have estimated fox population growth rates e.g., [17], [18], [40], [41], [42], those results have been presented as point estimates, with no indication of the confidence that could be placed in them. The temptation is, thus, to make comparisons between the growth rates of different populations, potentially attributing those differences to aspects of management or ecological circumstance. In this context, determining confidence intervals about estimates of λ is obviously essential, and our results highlight the need for caution in making comparisons between populations without accounting for uncertainty.

Knowledge of optimal sample sizes has implications for allocating resources, e.g. sampling effort for capture-mark-recapture studies. Our results indicate that, for red foxes, small initial increases in sample size will yield substantial reductions in uncertainty; however, as sample sizes increase, further effort to collect additional samples yields diminishing returns explained by a simple power law. Doak *et al.* (2005) suggest that it might often be beneficial to increase study duration, rather than sampling intensity. However, smaller sample sizes often lead to bias in demographic inference [7]. Our results suggest that small studies should be avoided but that, as sample size increases, it will be beneficial to devote resources towards determining the mechanistic basis for intrinsic variation, rather than simply to collect more samples; in many systems, this argues in favour of extending study duration to capture the drivers of inter-annual variation, commonly a significant source of variance.

To derive the relationship between sample size and uncertainty in λ, we assumed that individuals would be sampled approximately in proportion to the SSD. For studies based on mortalities such as shooting or road deaths, this seems to be an appropriate approach, assuming that the population approximates the SSD, but see [9]. In addition, studies that have considered the best allocation of sampling effort by age or stage e.g., [7], [43] have shown that sampling in proportion to the SSD is the approach likely to yield the least uncertainty in demographic parameters. Certainly, sampling in proportion to the SSD will yield a higher number of juveniles, which typically make the most significant contribution to fox population growth i.e. have the highest elasticities [18], [32]. Owing to the fact that the most important observation errors will arise from inadequate sampling of life stages with the highest elasticities [44], the value (in this case) of sampling in proportion to the SSD is clear. Although it is not possible to define a one-size-fits-all sampling intensity, the simple approach that we present should be applicable to a wide range of species. Moreover, the finding that quadrupling the sample size will typically halve the confidence interval is likely to be very general.

We have presented a brief example of how more information can be extracted from the type of published data that form a common source for demographic modelling. Our results highlight the fact that, even for well-studied species such as the red fox, sampling limitations and inherent variability can limit the precision with which we can identify characteristics of population dynamics. We recommend a more widespread use of these straightforward approaches (and related techniques), in order to promote a greater awareness of the limitations of many population analyses.

## Supporting Information

Figure S1 Likelihood distributions for vital rates simulated with varying sample sizes. Average survival rates of 0.1, 0.5, and 1.0 are simulated with varying age class sample sizes: (a–c) N = 10, (d–f) N = 100. All likelihoods were rescaled to peak at 1.0.
(0.22 MB DOC)

Table S1 Results of a literature review showing the number of studies that failed to include an accompanying measure of uncertainty of the estimated population growth rate. We conducted a Web of Science (http://apps.isiknowledge.com) search from January 2008 to May 2010 using the search terms ‘population growth’, ‘matrix model’, and ‘demography’. We separated the results by taxa, and further distinguished those that used previously published data to estimate matrix transition elements. We also recorded the impact factor of the journal for each result. The results are presented as a percentage of the total studies, the number of studies using published demographic data, and those published in a journal with a 5-year impact factor of four or higher (based on Web of Science, Journal Citation Reports).
(0.04 MB DOC)
