# Supplementary figures and images for: Uncertainty in Population Growth Rates: Determining Confidence Intervals from Point Estimates of Parameters
Source: PLoS One. 2010 Oct 25;5(10):e13628. doi: 10.1371/journal.pone.0013628 (PMC2963614; doi:10.1371/journal.pone.0013628)

Figure S1.


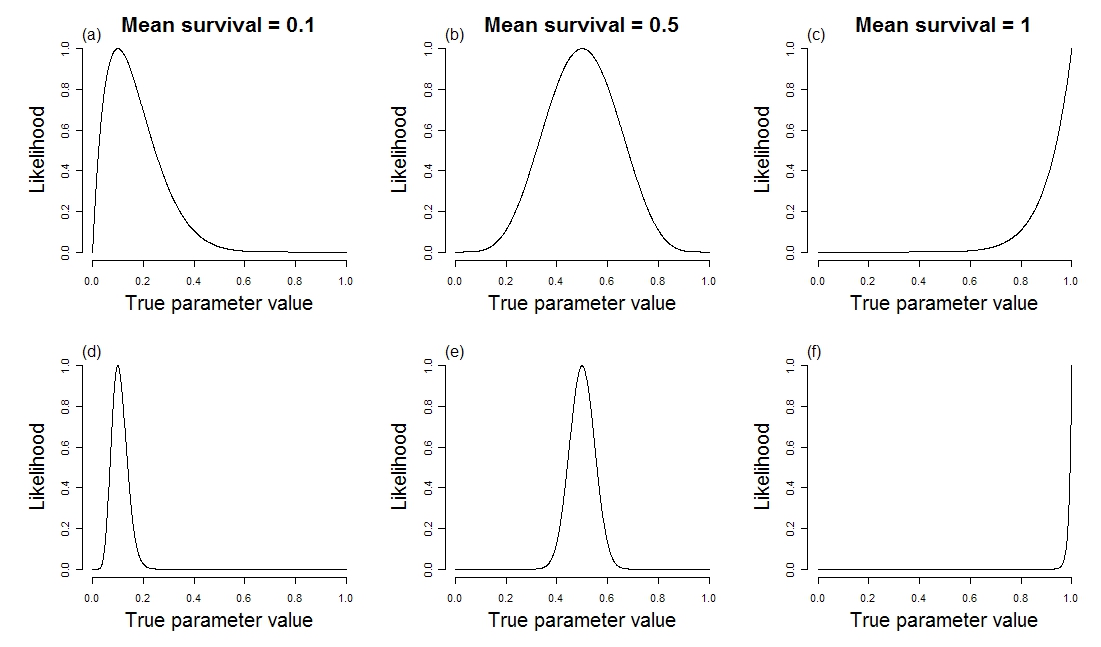

Supplement: Figure S1 — Likelihood distributions for vital rates simulated with varying sample sizes. Average survival rates of 0.1, 0.5, and 1.0 are simulated with varying age class sample sizes: (a–c) N = 10, (d–f) N = 100. All likelihoods were rescaled to peak at 1.0. (0.22 MB DOC) [file pone.0013628.s001.doc]
