# Supplementary material for: Uncertainty in Population Growth Rates: Determining Confidence Intervals from Point Estimates of Parameters
Source: PLoS One. 2010 Oct 25;5(10):e13628. doi: 10.1371/journal.pone.0013628 (PMC2963614; doi:10.1371/journal.pone.0013628)
Supplement: Table S1 — Results of a literature review showing the number of studies that failed to include an accompanying measure of uncertainty of the estimated population growth rate. We conducted a Web of Science (http://apps.isiknowledge.com) search from January 2008 to May 2010 using the search terms ‘population growth’, ‘matrix model’, and ‘demography’. We separated the results by taxa, and further distinguished those that used previously published data to estimate matrix transition elements. We also recorded the impact factor of the journal for each result. The results are presented as a percentage of the total studies, the number of studies using published demographic data, and those published in a journal with a 5-year impact factor of four or higher (based on Web of Science, Journal Citation Reports). (0.04 MB DOC) [file pone.0013628.s002.doc]

Table S1. Results of a literature review to assess the prevalence of confidence reporting in studies of population growth rates

| Taxon | Studies without | Studies using | Studies using published | Studies with | Studies without CIs |
| --- | --- | --- | --- | --- | --- |
|  | confidence | published | rates with no | 5-year impact | with 5-year |
|  | estimates (%) | vital rates (%) | confidence estimates (%) | factor ≥ 4 (%) | impact factor ≥ 4 (%) |
|  |  |  |  |  |  |
| Birds | 58 (19) | 37 (19) | 57 (7) | 21 (19) | 27 (11) |
| Fish | 70 (10) | 60 (10) | 67 (7) | 50 (10) | 43 (7) |
| Herptiles | 17 (6) | 0 (6) | 0 (0) | 17 (6) | 0 (1) |
| Insects | 43 (7) | 43 (7) | 67 (3) | 57 (7) | 33 (3) |
| Mammals | 38 (26) | 23 (26) | 50 (6) | 54 (26) | 50 (10) |
| Plants | 31 (35) | 9 (35) | 100 (3) | 17 (35) | 73 (11) |
| Other | 50 (6) | 17 (6) | 100 (1) | 45 (6) | 0 (3) |
| Total | 42 (109) | 24 (109) | 65 (26) | 45 (109) | 43 (46) |
